# All-trans retinoic acid suppresses the angiopoietin-Tie2 pathway and inhibits angiogenesis and metastasis in esophageal squamous cell carcinoma

**DOI:** 10.1371/journal.pone.0174555

**Published:** 2017-04-03

**Authors:** Na Li, Yanjuan Lu, Daoming Li, Xiangyu Zheng, Jingyao Lian, Shanshan Li, Huijuan Cui, Linda Zhang, Luqian Sang, Ying Wang, Jane J. Yu, Taiying Lu

**Affiliations:** 1 Department of Oncology, The First Affiliated Hospital of Zhengzhou University, Zhengzhou, Henan Province, People's Republic of China; 2 Department of Pathology, The First Affiliated Hospital of Zhengzhou University, Zhengzhou, Henan Province, People's Republic of China; 3 School of Life Sciences, Zhengzhou University, Zhengzhou, Henan Province, People's Republic of China; 4 Department of Oncology, Zhengzhou First People’s Hospital, Zhengzhou, Henan Province, People's Republic of China; 5 Department of Oncology, The Chinese People’s Liberation Army 150 Central Hospital, Luoyang, Henan Province, People's Republic of China; 6 Department of Behavioral Neuroscience, Northeastern University, Boston, MA, United States of America; 7 Division of Pulmonary, Critical Care and Sleep Medicine, University of Cincinnati College of Medicine, Cincinnati, OH, United States of America; Laboratoire de Biologie du Développement de Villefranche-sur-Mer, FRANCE

## Abstract

Esophageal squamous cell carcinoma (ESCC) is the second common cancer in Henan province and is well-known for aggressiveness and dismal prognosis. Adjuvant therapies, chemotherapy, radiotherapy and endoscopic treatment have not improved survival rates in patients with late stage esophageal carcinoma. All-trans retinoic acid (ATRA) is the active ingredient of Vitamin A and affects a wide spectrum of biological processes including development, growth, neural function, immune function, reproduction, and vision. It is one of the most potent therapeutic agents used for treating cancers, especially lung adenocarcinomas. ATRA inhibits metastatic potential and angiogenesis in several tumor models. We investigated the effects of ATRA on the expression of angiopoietin 1 (Ang-1), angiopoietin 2 (Ang-2) and receptor Tie-2 in EC1 cells in vitro. We also assessed the growth and migration of EC1 cells in vitro. ATRA treatment caused 29.5% and 40.3% reduction of the growth of EC1 cells after 24 hours and 48 hours, relative to the control. ATRA plus fluorouracil treatment reduced the viability more strongly than either drug alone, indicating an additive effect. Moreover, ATRA decreased EC1 migration by 87%. Furthermore, ATRA treatment led to a marked decrease of the transcript levels of Ang-1, Ang-2, Tie-2, VEGF, and VEGF receptors, as assessed by real-time RT-PCR. Importantly, the protein levels of Ang-1, Ang-2 and Tie-2 were reduced by ATRA treatment. In vivo, we found ATRA treatment suppressed the tumor growth and improved the cachexia of mice. Importantly, ATRA treatment decreased the expression of CD31, Ang-1, Ang-2 and Tie-2 in subcutaneous tumors of EC1 cells. Collectively, our findings demonstrate that ATRA exhibits a dose- and temporal-dependent effect on the metastatic behavior, suppresses the angiopoietin-Tie2 pathway and inhibits angiogenesis and the progression of xenograft tumors of EC1 cells.

## Introduction

Esophageal cancer is one of the five most commonly diagnosed cancers in humans. It is the third most common diagnosed malignancy and the fourth leading cause of death in China. In northern China, esophageal cancer has become the leading cause of cancer-related death due to the highly aggressive nature of the cells, high incidence in this region, and in diagnosis. These delays lead to later staging levels at diagnosis. Drugs are based on the aggressiveness of the tumors, and plays an integral part in guiding stage specific treatment protocols; stage at diagnosis has a real impact on overall patient survival[1–3]_._ There is no doubt that the patient accepted treatment early, then proper drugs and appropriate adjuvant therapy, such as radiotherapy and endoscopic treatment, can reduce the mortality from esophageal cancer[4, 5]. Although the number of long-term survivors who have received chemo radiation therapy is increasing, the molecular mechanisms of some drugs are still unknown. It is important to discover and understand the molecular events underlying the initiation, proliferation, migration and metastasis of esophageal cancer.

Experimental evidence identifies angiogenesis as one of the important factors in malignant tumor recurrence and metastasis. Tumor progression requires the formation of new blood vessels or angiogenesis mimicry to maintain the blood supply of the tumor cells[6, 7]. The vascular endothelial growth factor (VEGF) pathway, angiopoietins and their Tie receptors play central roles in tumor angiogenesis, tumor cell proliferation, migration, and angiogenesis mimicry and extracellular matrix degradation[7, 8]. Tie-2 is of great importance for vascular maturation during developmental, physiological and pathological angiogenesis. Angiopoietin 1–4 (Ang-1–4) have been identified as ligands of the Tie-2 receptor[9–10]. Ang-1 and Ang-2 have been studied extensively. Ang-1 plays a critical role in cell survival, adhension, migration and angiogenesis. Ang-2 inhibits development of vessels and angiogenesis, and also promotes cell death. However, VEGF, together with Ang-2 promotes development of new vessels. Hence, angiopoietins play crucial roles in the angiogenic switch during the development of tumor. When the tumor develops quickly, which means poor prognosis, there will be more expression of Angiopoietins, especially Ang-1 and Ang-2. The role angiopoietins play in physiological and pathological angiogenesis makes it be a promising target in the vascular diseases and cancer treatment[11]. Although we have gained partial success in tumor treatment, such as anti-VEGF therapy, a variety of mechanisms remains a major obstacle. The search for novel key downstream effectors such as Angiopoietins-Tie and other known growth factors, which may have potential significance in the perspective of angiogenesis control in cancer, is essential to develop treatments for esophageal cancer. Angiopoietins -Tie2 is a major driver of tumor growth, but it is still unknown whether it plays any role in the progression of esophageal cancer[12–14].

All-trans retinoic acid (ATRA), a naturally occurring metabolite of vitamin A, has great potential as an antitumor genic drug to treat acute leukemia by promoting cancer cell differentiation[15]. ATRA as one kind of hormonal retinoids, can regulate target gene expression by the activation of two kinds of nuclear retinoic receptors: RAR and RXR[16]. Studies also have found that ATRA can inhibit esophageal cancer cell proliferation and migration, and may be associated with inhibition of VEGF-related tumor angiogenesis. It is known that tumor blood supply is dependent on angiogenesis and antigenic mimicry, but the role of cytokines and their related signaling pathways is not clear; there may be multiple signaling pathways or multiple targets[17, 18]. In this study, we have found the effect of ATRA on proliferation and migration of the esophagus carcinoma cell line EC1 in vitro. ATRA treatment also decreases the expression of Ang-1, Ang-2, Tie-2, VEGF and VEGF receptors in EC1 cells. ATRA and Fluorouracil showed additive effect on both cell proliferation and the genes expression of Angiopoietins -Tie2 pathway in vivo. ATRA treatment suppresses the tumor growth and improved the cachexia of mice. ATRA also decreased the expressions of CD31, Ang-1, Ang-2 and Tie-2 in subcutaneous tumors. Our study provides the proof-of-concept of a novel anti-angiogenesis therapeutic strategy for patients with esophageal cancer.

## Materials and methods

### Compounds and reagents

ATRA (anhydrous ethanol mixture 1000 μg/ml stock solution, 4°C) was purchased from Sigma (USA). Fluorouracil (5-Fu) was purchased from Haipu Pharmaceutical Factory (Shanghai, China). AM80 was purchased from Abcam (MA, USA). RPMI1640 medium was purchased from Solarbio (Beijing, China). Fetal bovine serum was purchased from Sijiqing (Hangzhou, China). CCK-8 was purchased from Dojindo Molecular Technologies, Inc. (Kumamoto, Japan). Rabbit anti-Angiopoietin 1 antibody, rabbit anti-Angiopoietin 2 antibody, and rabbit anti-Tie-2 antibody were purchased from Abcam (MA, USA). Rabbit anti- Cleaved caspase3 antibody, Mouse anti-Cleaved PARP antibody, Mouse anti-β-actin antibody were purchased from Cell Signaling Technology (MA, USA). RNeasy Mini Kit was purchased from QIAGEN (USA). High-capacity cDNA reverse transcription Kit was purchased from Applied Biosystems (Branchburg, NJ, USA). SYBR were bought from Beijing kangcheng biological technology (Beijing China). Oligonucleotides were purchased from Shanghai biological engineering technology service co.; LTD. Super signal West Pico Chemiluminescent Substrate was purchased from Thermo Fisher Science (USA).

### Cell lines and cell culture

Human esophageal cancer cell line, EC1, was given by The Medical College of Zhengzhou University. EC1 single-layer adherent cells were grown in RPMI1640 medium containing 10% fetal bovine serum, and incubated at 37°C and 5% CO2. Experiments were conducted when cells were in the exponential growing phase, as assessed by light microscopy.

### Cell proliferation assay

Cell viability was determined using the Cell Counting Kit-8 (CCK-8; Dojindo Molecular Technologies, Kumamoto, Japan) according to the manufacturer's instructions. A 100 mL suspension of EC1 was plated at a density of 5 x10^4^ cells/mL in 96-well plates, and incubated overnight. The following day, fresh media supplemented with 10% FBS. Cells were treated with ATRA, Fluorouracil, ATRA plus Fluorouracil, or no treatment control for 24 and 48 hours. 10 μL of CCK-8 solution was added to each well, incubated for one-hour, and the optical density (OD) value was measured at 450 nm using Spectrophotometer NANADROP2000 (Thermo Scientific, USA). The cell viability was calculated as follows: Cell Viability [%] = [Abs (sample)-Abs (blank)/Abs (Negative control)-Abs (blank)] x 100.

### Wound healing assay

EC1 cells were seeded at a density of 5×10^5^ cell in 6-well plates and incubated for 24 hours. A sterile 200-μl pipette tip was used to scratch the cell layer, making a clear line. The cells were then rinsed with phosphate-buffered saline (PBS) to remove cells not attached to the dish, and then cultured in serum-free RPMI1640 under different conditions (0.1, 1, and 10 μmol/L ATRA, 100 mg/L Fluorouracil and no treatment as control) for 24 hours. Using a digital camera system, images were captured at 24 hours after the scratches were made. The experiments were performed in triplicate and repeated at least 3 times.

### Gene expression analysis

Real-time quantitative (RT-qPCR) was carried out to analyze gene expression analysis. Total RNA was prepared from EC1 cells using RNeasy Mini Kit according to the manufacturer's instructions; RNA concentrations were determined using Gen 5. First strand cDNA was synthesized from 2μg of total RNA of each cell sample using a High-capacity cDNA reverse transcription Kit (Applied Biosystems, Branchburg, NJ, USA) according to the manufacturer's instructions. The complementary DNAs (cDNA) were then used as templates for real-time qPCR with SYBR® Select Master Mix (Applied Biosystems, USA. Primer sets we used are as follows:

GAPDH: Forward:5’-GGAGATTACTGCCCTGGCTCCTA-3’,

Reverse: 5’-GACTCATCGTACTCCTGCTTGCTG-3’.

Ang-1: Forward: 5’-AGCGCCGAAGTCCAGAAAC-3’,

Reverse: 5’-TACTCTCAGACAGTTGCCAT-3’.

Ang-2: Forward: 5’-AACTTTCGGAAGAGCATGGAC-3’,

Reverse: 5’-CGAGTCATCGTATTCGAGCGG-3’.

Tie-2: Forward: 5’-TTAGCCAGCTTAGTTCTCTGTGG-3’,

Reverse: 5’-AGCATCAGATACAAGAGGTAGGG-3’,

VEGF: Forward: 5’- TGGTGTCTTCACTGGATGTATTT-3’,

Reverse: 5’- GAAGAGGAGGAGATGAGAGACT-3’,

Flt: Forward: 5’-AACGCATAATCTGGGACAGTAG-3’,

Reverse: 5’-AAATAGGGCTTCTGACCTGTG-3’,

KDR: Forward: 5’-AGCAGGATGGCAAAGACTAC-3’,

Reverse: 5’-CTGTATGGAGGAGGAGGAAGTA-3.’ Quantitative PCR analysis was performed using the Quant Study Design Analysis System v 1.0 in QuantStudio3 (BIO-RAD, USA). GAPDH was used for normalization of expression data. Fold change = 2^−ΔΔCt,^ ΔΔCt = (CT Sample–Ct GAPDH)–(Ct Control–Ct GAPDH).

### Western blot analysis

Cells were washed with PBS, then lysed with RIPA lysis buffer supplemented with 100 x protease inhibitors, 100 x phosphatase inhibitors and 0.1 M PMSF (Abcam, USA). Cell lysates were then harvested by centrifugation at 14000 rpm for 5 minutes, at 4°C. Protein extracts in the supernatant were transferred into new tubes, and protein concentration was measured by BCA protein assay method. Proteins were analyzed using 10% SDS-PAGE gel electrophoresis, and then transferred onto a PVDF membrane. The membrane was incubated in 5% skim milk in TBS-T for blocking at room temperature for 1 hour, washed with TBS-T three times for 15 minutes, and then incubated with primary antibody solution (1:1000 dilution for all antibodies) at 4°C over-night, followed by secondary antibody incubation for 3 hours at room temperature. The immuno-reactive signals were visualized with Super Signal West Pico Chemiluminescent Substrate, using G-BOX chemi-XRQ. Detected signals were quantified using Genesys. ß-actin proteins were shown as a loading control and used for quantification normalization.

### Animal studies

Female BALB/c nude mice (4–5 weeks of age) were purchased from Beijing Vital River Experimental Animal Technical Co., LTD (China). Mice were feed in SPF stage animal rooms in medical sciences Institute of Zhengzhou University. All procedures met the standards in the Guide for the Care and Use of Laboratory Animals. The protocol was approved by the Committee on the Ethics of Animal Experiments of the First Affiliated Hospital of Zhengzhou University (Permit Number: 2015[5]). We randomized 25 mice into five treatment groups to replicate the in vitro experiments above. 1×10^6^ EC1 cells were subcutaneously inoculated into the right suprascapular regions of BALB/c nude mice. Once tumors became visible, mice were randomized and treated for 10 days with ATRA (0.1, 1, or 10 mg/kg/day by gavage), Fluorouracil (50 mg/kg/day), or placebo (DMSO or PBS). We anesthetized animals with isoflurane during cell inoculation. We monitored the development of subcutaneous tumors daily and recorded body weight (grams) weekly. Tumor size was calculated: width×length. The endpoint of the xenograft tumor study was the onset of the clinical signs of pain/distress including 1) animals are in constant pain (hunched posture, sluggish movement, vocalization when handled); 2) bilateral tumors have caused inactivity, became ulcerated and/or larger than 15% of the animals’ body weight; 3) animals have lost more than 20% of their body weight. All mice were euthanized by Cervical Dislocation. We checked the expression of Ang-1, Ang-2 and Tie-2 in xenograft tumor tissues by Immunological Histological Chemistry (IHC).

### Statistical analysis

The statistical analysis of the data was performed using Student’s-t test when two groups were compared, using Graph Pad Prism 5 software. Results are presented as mean±SD. For all tests, p < 0.05 was considered statistically significant.

## Results

### ATRA inhibits EC1 cell proliferation in vitro

**Fig 1:**
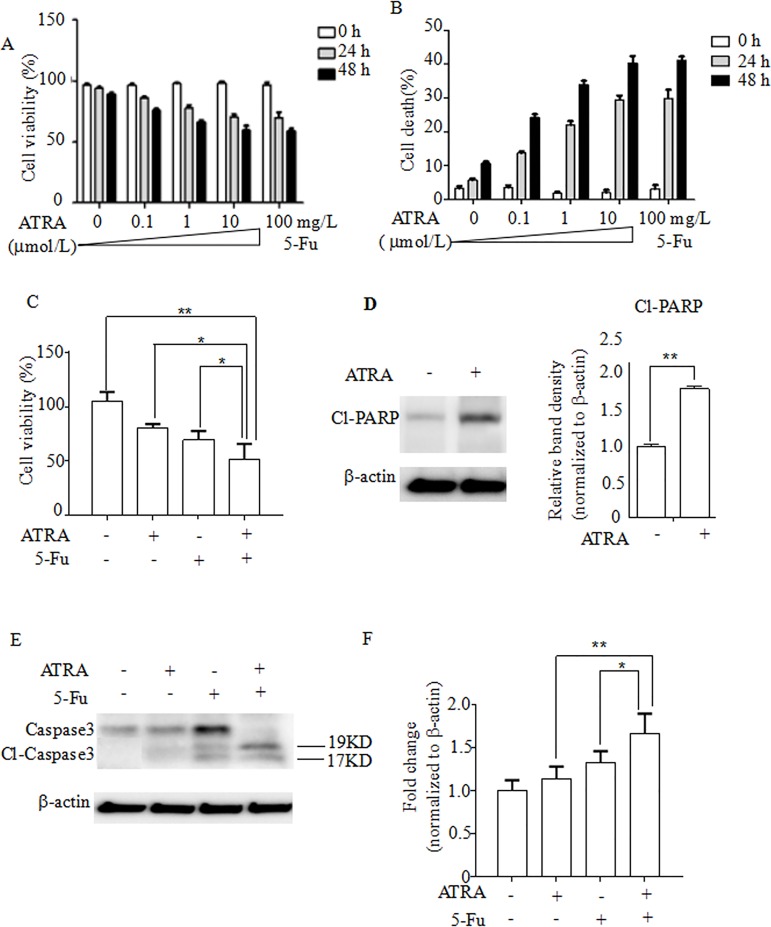
ATRA treatment shows a time-dependent and dose-dependent inhibition on cell viability. EC1 cells were cultured in RPMI-1640 supplemented with 10% FBS and seeded in 96 well plates. (A) After 0 hours, 24 hours and 48 hours, the number of live cells was counted in ATRA and fluorouracil treatment groups. (B) The proportion of dead cells were calculated and presented as Percent of death cells/total number of cells. (C) After 24 hours, ATRA and fluorouracil show additive effect on cell proliferation. (D) After 24 hours, ATRA caused apoptosis of cells. Densitometry analysis of protein levels of Cl-PARP. (E) The effect of ATRA and fluorouracil on the expression of Cl-Caspase3. (F) Densitometry analysis of protein levels of Cl-caspase3. *p<0.05; **p<0.01; *** p<0.001. Student t-test.

To examine the effects of ATRA on EC1 cell proliferation, cells were cultured for 24 and 48 hours in media including 0.1, 1, or 10 μmol/L ATRA, Fluorouracil (100 mg/L) or no treatment. Cells incubated in 0.1μmol/L ATRA showed 13.7% and 24.2% reduction at 24 and 48 hours, relative to the control, respectively (p < 0.05; **Fig 1A**). Cells incubated in 1 μmol/L ATRA showed 22.0% and 33.9% reduction at 24 hours and 48 hours, relative to untreated cells, respectively (p < 0.05; **Fig 1A**). Cells incubated in 10 μmol/L ATRA showed 29.5% and 40.3% reduction at 24 hours and 48 hours, relative to untreated cells. 100 mg/L Fluorouracil caused 29.9% and 41.2% reduction at 24 hours and 48 hours, relative to untreated cells, respectively (p < 0.05; **Fig 1A**). The difference between the reductions caused by 10μmol/L ATRA and 100 mg/L Fluorouracil were not significant (p > 0.05; **Fig 1A**). We also calculated the dead cell number in each conditions. Cells incubated in 0.1 μmol/L ATRA showed 1.24 and 1.18 times increase of dead cells at 24 and 48 hours, relative to untreated cells, respectively (p < 0.05; **Fig 1B**). Cells incubated in 1 μmol/L ATRA showed 2.28 and 2.9 times increase of dead cells at 24 and 48 hours, relative to untreated cells, respectively (p < 0.05; **Fig 1B**). Cells incubated in 10 μmol/L ATRA showed 3.17 and 2.45 times increase of dead cells at 24 and 48 hours, relative to untreated cells, respectively (p < 0.05; **Fig 1B**). 100 mg/L Fluorouracil caused 3.19 and 2.47 times increase of dead cells at 24 and 48 hours, relative to untreated cells, respectively (p < 0.05; **Fig 1B**). Next, we examined the ATRA effect on EC1 apoptosis. 10 μmol/L ATRA plus 100 mg/L Fluorouracil caused 42.3% reduction of cell viability after 24 hours’ treatment, while ATRA alone caused 21.5% reduction and Fluorouracil alone caused 25.2% reduction, relative to untreated cells, respectively (p < 0.05; **Fig 1C**), suggesting that ATRA promotes cell death. Moreover, immunoblotting analysis showed that ATRA treatment increased the level of cleaved-PARP by 82% compared with the control (p < 0.05; **Fig 1D**), indicating that ATRA induces the apoptosis of EC1 cells. After 24 hours of ATRA plus Fluorouracil treatment, we checked the expression of Cleaved caspase3 (**Fig 1E**). We compared ATRA treatment, fluorouracil treatment together with ATRA plus fluorouracil treatment. After 24 hours’ treatment, ATRA caused 13.5% increase of the Cl-caspase3 expression, and Fluorouracil caused 33.2% increase, while ATRA plus Fluorouracil caused 66.9% increase, relative to the control, respectively (p < 0.05; **Fig 1F**).

### ATRA inhibits cell migration as measured by wound healing assay

**Fig 2:**
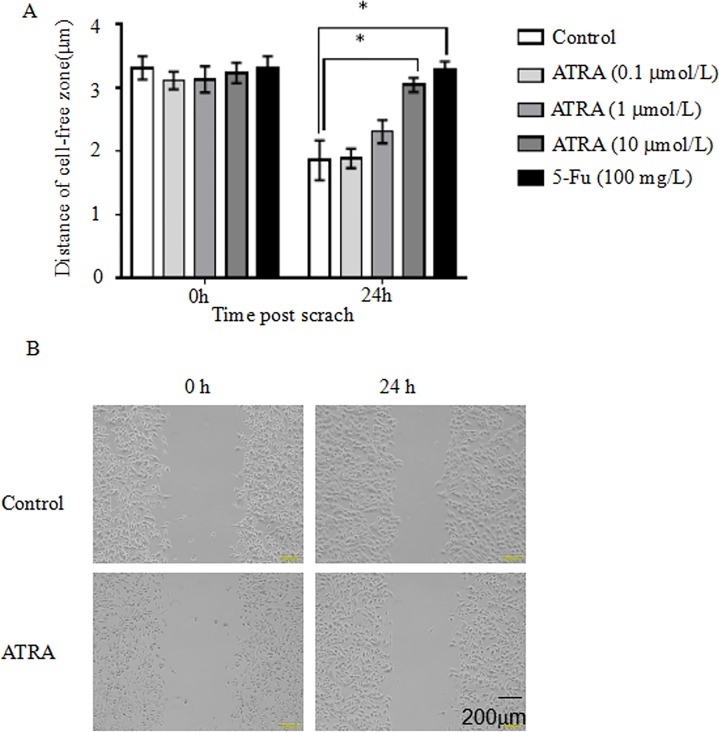
ATRA treatment reduces cell migration in EC1 cells. EC1 cells were cultured in RPMI-1640 supplemented with 10% FBS and seeded in 6 well plates. Scratches on cell monolayer were made using pipette tips when cells became confluent. Cells were then treated with 3 concentrations of ATRA (0.1, 1, 10 μmol/L), fluorouracil (100 mg/L), or untreated for 24 hours. Images were chosen from 10 random fields to calculate the average distances. Data were presented as average length of cell-free void ± SD. (B) Representative pictures of wound healing assay. *p<0.05; **p<0.01; *** p<0.001. Student t-test.

To study the effect of ATRA inhibition on EC1migration, wound healing assays were performed under the same conditions as above, with images captured at 24 hours’ post wound. We selected 10–15 points at the edge of cells and used the average distance to compare. After 24 hours’ treatment, the distance of no treatment was 1.457 μM. The distance of 0.1μmol/L ATRA was 1.227 μM. The distance of 1 μmol/L ATRA was 0.825 μM. The distance of 10 μmol/L ATRA was 0.188 μM. The distance of 100 mg/L Fluorouracil was 0.046 μM. Compared with no treatment, the distance between the cells in 0.1 μmol/L ATRA has decreased 15.8%, the distance between the cells in 1 μmol/L ATRA has decreased 43.4%, the distance between the cells in 10 μmol/L ATRA has decreased 87.1%, the distance between the cells in 0.1 μmol/L ATRA has decreased 96.9%. The distances between the wound scratched in the cells were wider with increasing doses of ATRA, and especially 10 μmol/L ATRA and 100 mg/L Fluorouracil were significantly different from the no treatment control (**Fig 2A**) (p < 0.05).

### Gene expression of Ang-1, Ang-2, and Tie-2, VEGF and VEGF receptors was suppressed by ATRA and Fluorouracil

**Fig 3:**
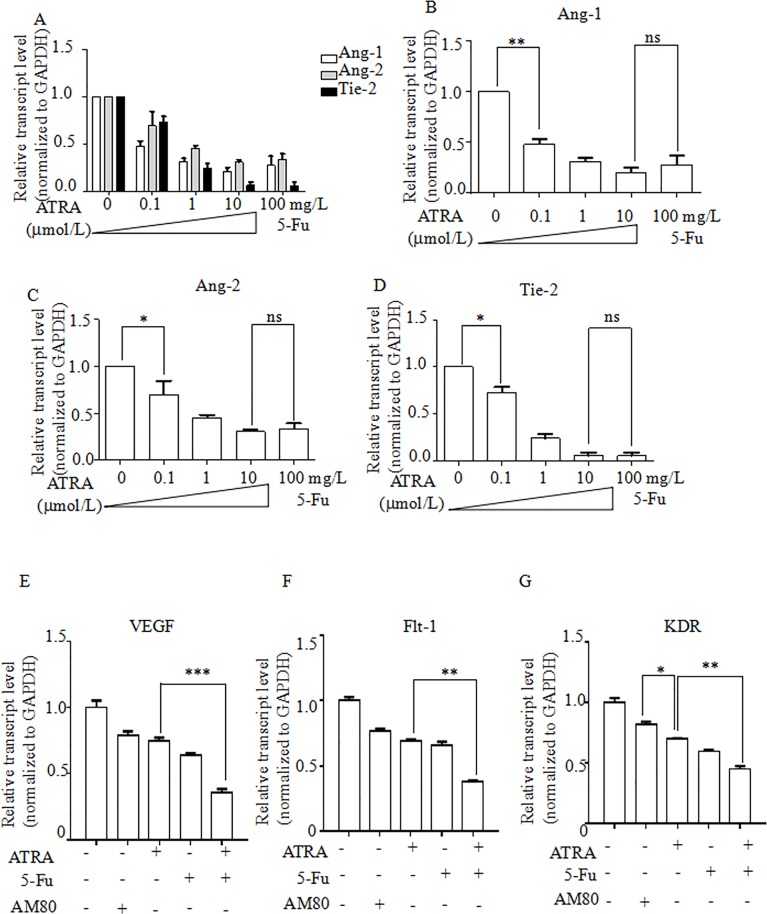
The transcript levels of Angiopioteins-Tie-2 pathway are downregulated in EC1 cells. EC1 cells were treated with ATRA at 0.1, 1, or 10 μmol/L, 100 mg/L fluorouracil, 10 μmol/L AM80, 100 mg/L fluorouracil plus 10 μmol/L AM80, or untreated for 24 hours. RNA was isolated from treated cells. Real-time RT-PCR analysis was performed to assessed the transcript levels of (A) Ang-1, Ang-2 and Tie-2. (B) Ang-1. (C) Ang-2. (D) Tie-2. (E) VEGF. (F) Flt-1. (G) KDR. *p<0.05; **p<0.01; *** p<0.001. Student t-test.

To investigate the effects of ATRA on the Angiopioteins-Tie2 pathway, we assessed DNA expression of Ang-1, Ang-2 and Tie-2 in EC1 cells incubated under the same conditions as above. After 24 hours’ treatment, DNA expression level was downregulated compared to controls in each condition (p< 0.05; **Fig 3**). We also found there are decrease in the expressions of Ang-1. The cells in 0.1 μmol/L ATRA had a decrease of 52%. The cells in 1 μmol/L ATRA had a decrease of 69%. The cells in 10 μmol/L ATRA had a decrease of 80%. The cells in 100 mg/L Fluorouracil had a decrease of 73% (**Fig 3B**). When it came to the expression of Ang-2, the cells in 0.1 μmol/L ATRA had a decrease of 30%. The cells in 1 μmol/L ATRA had a decrease of 55%. The cells in 10 μmol/L ATRA had a decrease of 69%. The cells in 100 mg/L Fluorouracil had a decrease of 66% (**Fig 3C**). Furthermore, the expression of Tie-2, the cells in 0.1 μmol/L ATRA had a decrease of 27%. The cells in 1 μmol/L ATRA had a decrease of 76%. The cells in 10 μmol/L ATRA had a decrease of 94%. The cells in 100 mg/L Fluorouracil had a decrease of 94% (**Fig 3D**). We checked the expression of VEGF, the cells in 10 μmol/L AM80 had a decrease of 20.8%. The cells in 10 μmol/L ATRA had a decrease of 25.3%. The cells in 100 mg/L Fluorouracil had a decrease of 36.3%. The cells in ATRA and Fluorouracil dual treatment had a decrease of 64.4%, relative to untreated cells, respectively (p < 0.05; **Fig 3E**). We checked the expression of Flt-1, the cells in AM80 had a decrease of 22.9%. The cells in ATRA had a decrease of 31.1%. The cells in Fluorouracil had a decrease of 34.0%. The cells in ATRA and Fluorouracil dual treatment had a decrease of 62.0%, relative to untreated cells, respectively (p < 0.05; **Fig 3F**). We checked the expression of KDR, the cells in AM80 had a decrease of 18.0%. The cells in ATRA had a decrease of 30.0%. The cells in Fluorouracil had a decrease of 40.2%. The cells in ATRA and Fluorouracil dual treatment had a decrease of 55.0%, relative to untreated cells, respectively (p < 0.05; **Fig 3G**).

### ATRA negatively regulates the expression of Ang-1, Ang-2 and Tie-2

**Fig 4:**
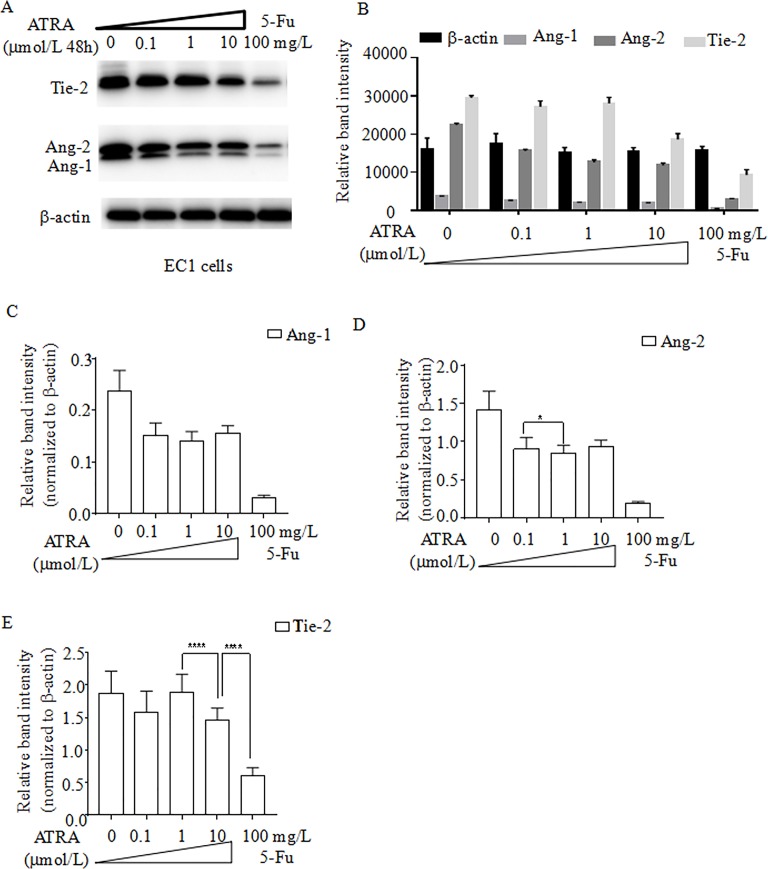
ATRA treatment decreases the expression of Ang-1, Ang-2 and Tie-2 in EC1 cells. EC1 cells were treated with 3 concentrations of ATRA (0.1, 1, 10 μmol/L), fluorouracil (100 mg/L) for 24 hours, or untreated. (A) The protein levels of Ang-1, Ang-2 and Tie-2 were examined using western blot. Densitometry analysis of the protein levels of Ang-1, Ang-2 or Tie-2 (B); Ang-1 (C); Ang-2(D); and Tie-2 (E). β-actin was used as a loading control. *p<0.05; **p<0.01; *** p<0.001. Student t-test.

To assess the effects of ATRA and fluorouracil on the angiopoietin-Tie2 pathway in EC1 cells, Ang-1, Ang-2 and Tie-2 protein expression was assessed by western blot under the conditions above (**Fig 4A**). There was a trend of decline of Ang-1, Ang-2 and Tie-2 protein expression with increasing dose of ATRA, and also with 100 mg/L fluorouracil treatment (**Fig 4B**). Compared the expression of Ang-1 with no treatment, the cells in 0.1 μmol/L ATRA had a decrease of 36%. The cells in 1 μmol/L ATRA had a decrease of 40%. The cells in 10 μmol/L ATRA had a decrease of 35%. The cells in 100 mg/L Fluorouracil had a decrease of 87% (**Fig 4C**). Compared the expression of Ang-2 with no treatment, the cells in 0.1 μmol/L ATRA had a decrease of 36%. The cells in 1 μmol/L ATRA had a decrease of 40%. The cells in 10 μmol/L ATRA had a decrease of 35%. The cells in 100 mg/L Fluorouracil had a decrease of 87% (**Fig 4D**). Compared the expression of Tie-2 with no treatment, the cells in 0.1 μmol/L ATRA had a decrease of 15%. The cells in 1 μmol/L ATRA had a decrease of 0.6%. The cells in 10 μmol/L ATRA had a decrease of 22%. The cells in 100 mg/L Fluorouracil had a decrease of 68% (**Fig 4E**).

### ATRA can reduce the tumor load and improve cachexia

**Fig 5:**
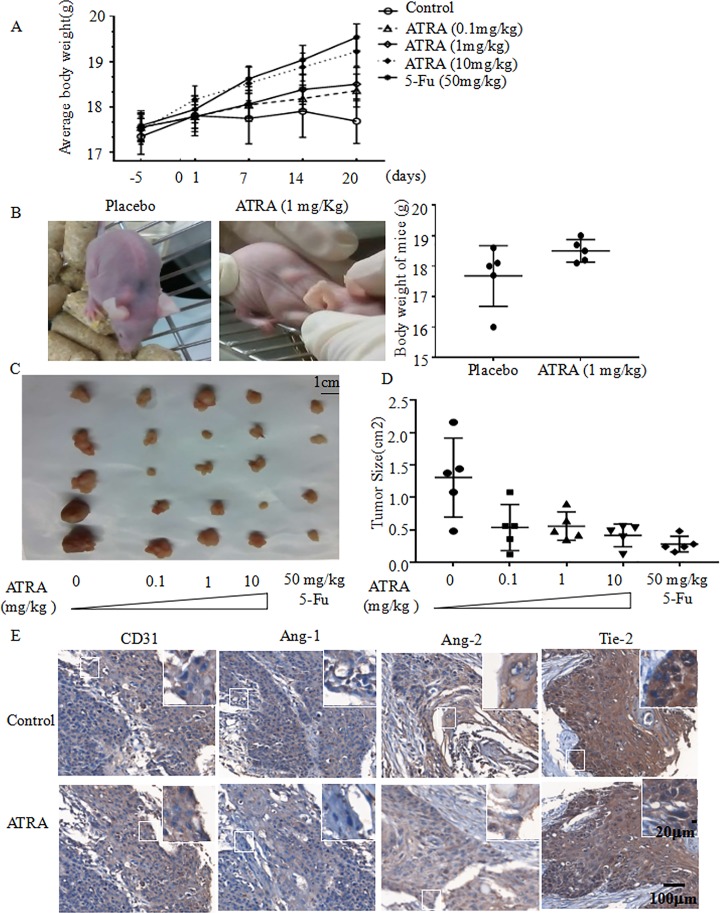
ATRA treatment suppresses the growth of xenograft tumors of EC1 cells and improves the cachexia of mice. (A) 1x10^6^ EC1 cells were subcutaneously injected into mice at both flanks on day 0. Ten days post-cell inoculation, mice bearing xenograft tumors were randomized to five groups and treated for 10 days with placebo, fluorouracil (50 mg/kg/day), or 3 concentrations of ATRA (0.1, 1, or 10 mg/kg/day). Mice were killed on day 20. Mouse body weight was measured before and after cells implantation, also before and after treatment. (B) The cachexia was recorded in mice treated with ATRA, fluorouracil, or placebo. Cachexia was assessed by body weight loss. (C) Images of tumors isolated from mice treated with ATRA and fluorouracil. (D) Average tumor size was calculated and shown in panel C. (E) Immunohistochemical staining of CD31, Ang-1, Ang-2 and Tie-2 in subcutaneous tumors. *p<0.05; **p<0.01; *** p<0.001. Student t-test.

Pre-clinical studies were performed to assess the effects of ATRA on EC1 cells in vivo. To evaluate the efficacy, Mice were injected subcutaneous with EC1 cells, and we divided 25 mice into five treatment groups to replicate the in vitro experiments above. When tumors became visible, mice were gavaged with different concentrations (0.1, 1, 10 mg/Kg) of ATRA, (50 mg/Kg/day) fluorouracil, or PBS and DMSO as placebo. We weighted the body weight of mice every week. After 10 days’ treatment, the body weight of 0.1 mg/Kg ATRA treatment group have 4% increase compared with no treatment. The body weight of 10 mg/Kg ATRA treatment group have 9% increase compared with no treatment (**Fig 5A**). Mice in the placebo group exhibited cachexia comparted to the treatment groups (**Fig 5B**). Mice in the treatment groups produced smaller tumors compared to control. ATRA can significantly decrease the area size of xenograft tumors. The average tumor size of no treatment are 2.2 times larger than 10 mg/Kg ATRA group. Compared with the tumor size of 10 mg/Kg ATRA group, 0.1 mg/Kg ATRA group has 30% increase and 0.1 mg/Kg ATRA group has 34% increase. When compared with 10 mg/Kg ATRA with 50 mg/Kg/day fluorouracil, there is a 50% increase (**Fig 5C**, **Fig 5D**). Next, we examined the expression of Ang-1, Ang-2 and Tie2 in subcutaneous tumors of EC1 cells (**Fig 5E**). We found that ATRA decreased the expression of Ang-1, Ang-2 and Tie-2. Importantly, we also found that CD31 immunoreactivity was reduced in xenograft tumors from ATRA-treated mice, indicating a mechanism of action for ATRA in tumor angiogenesis in vivo (**Fig 5E**).

## Discussion

Angiogenesis is a process of new blood vessel development from the preexisting vascular network[19]. Vessels are the key features of cancer progression, and they offer abundant oxygen and nutrition to make sure tumors can have enough materials to grow. The balance of proangiogenic growth factors such as VEGF and inhibitors can induce angiogenic phenotypes. Active angiogenesis has been shown to be an essential process for new vessel formation, tumor growth, progression, and spread. Tumor blood vessels can become a treatment target point[20–22]. Therefore, inhibition and destruction of tumor blood vessels using tumor angiogenesis inhibitors (TAI) can reduce nutrition and "starve" tumors.

All-trans retinoic acid (ATRA), one of the vitamin A compounds, plays an important role regulating and controlling the growth and proliferation of metastasis. Our previous study found that both ATRA and fluorouracil can induce marked tumor repression. ATRA decreases the expression of caspase3, Bcl-2, inhibits the apoptosis signaling pathway, and also reduces the expression of VEGF and the component of the VEGF signal transduction pathway of CD31, CD34, and CD105 in vitro and in vivo [23–26]. According papers and our previous experiment, we inferred that VEGF may be a potential target for pharmacological intervention of esophageal squamous cell carcinoma.

After VEGF/VEGFR system was found, Tie receptors (including Tie—1 and Tie -2) and their ligands Angiopoietins (Ang1-4) were identified as members of the vascular tissue specificity tyrosine kinase system[27–28]. The VEGF-VEGFR Angiopoietin- Tie systems regulate different kinds of blood and lymphatic vessel growth. Thus, targeting both systems may be beneficial in maximizing the efficacy of anti/pro-angiogenic therapies[29]. Angiopoietin1–4 (Ang-1–4) are named for their function as crucial paracrine factors for tumor growth. Angiopoietin-1 can bind with Tie-2 as its receptor phosphorylate it. Angiopoietin-Tie-2 compound can promote blood vessel growth. Ang-2 acts as an antagonist to disrupt the Ang-1/Tie-2 protective signaling. Ang-2 also weakens the endothelial barrier function and enhances the endothelial responsiveness to stimulation by angiogenic cytokines, like vascular endothelial growth factor (VEGF) [30–32]. Studies have shown that plasma Angiopoietin-2 increases after Non-small cell lung cancer (NSCLC) surgery and contributes to the proangiogenic property of the postoperative plasmas. This supports the possible administration of anti-Ang2 therapy for NSCLC in a postoperative adjuvant setting[33]. Therefore, if we want to control the growth of tumors, targeting Angiopoietins-Tie2 signaling, especially Ang-1, Ang-2, Tie-2, in esophageal cancer cells seems to be a promising and important mechanism for cancer treatment[34].

The aim of this study was to investigate the effect of low concentrations of all-trans retinoic acid (ATRA) on esophageal squamous carcinoma cells (ESCC) and explore potential mechanisms of action. Studies have shown that low- dose decitabine plus ATRA is a promising treatment for myeloid neoplasm patients with myeloid judged ineligible for intensive chemotherapy[35]. Some papers already proved that after fluorouracil treatment, there was a rapid increase in plasma vascular endothelial growth factor A (VEGF-A) and Tie-2 levels. Inhibition of Tie2 resulted in impaired neoangiogenesis, leading to a delay in hematopoietic recovery[36]. We wanted to know whether low concentrations of all-trans retinoic acid could inhibit the growth of vessels through the Angiopoietin1–4-Tie2 pathway.

If ATRA works, we also want to know the influence ATRA shows on EC1 cells, especially when compared with fluorouracil and dual with fluorouracil. These results can provide the rational for low concentration retinoid maintenance treatment for esophageal cancer in the clinical setting.

**Fig 6:**
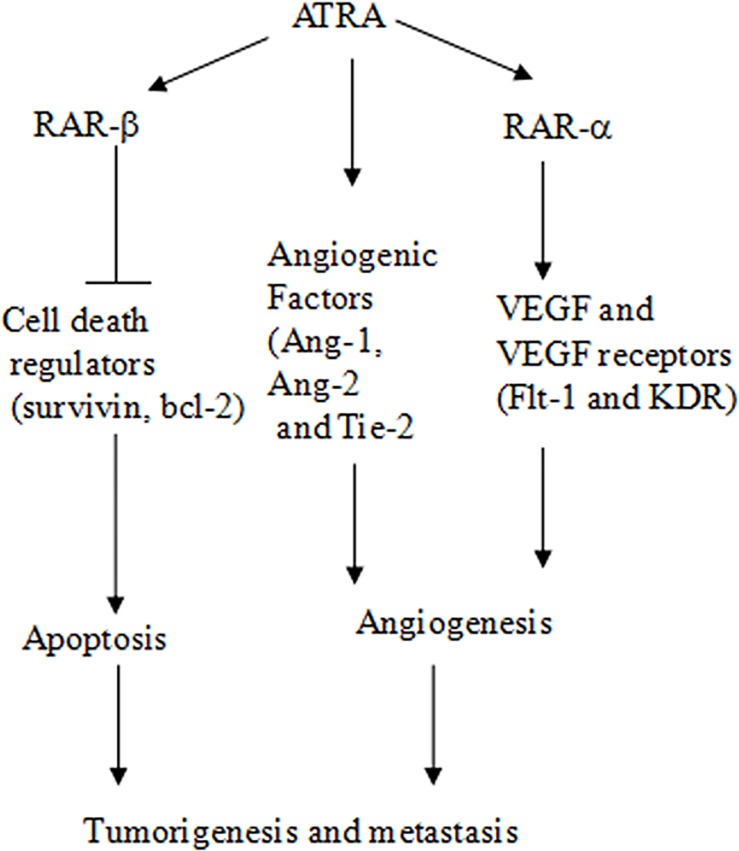
Schematic illustration of ATRA effect on cell viability and angiogenesis in esophageal carcinoma. ATRA treatment induces cell apoptosis by controlling cell death regulators (survivin and bcl-2) via RAR-β, inhibits VEGF-VEGFR signaling pathway via RAR-α, and regulates the expression of angiogenic factors (Ang-1, Ang-2 and Tie-2) and angiogenesis. In vivo, ATRA suppressed tumor growth and metastasis. Previously reports and the current study demonstrate that ATRA inhibits cell viability, angiogenesis and metastasis of esophageal cancer cells in vitro and in vivo.

We previously reported that ATRA treatment decreased the expression of vascular endothelial growth factors including VEGF, CD34, and CD105 in EC9706 in vitro and in vivo. We also found that ATRA induced caspase-3 cleavage and reduced the levels of anti-cell death protein Bcl-2 and survivin via RARβ, associated with suppressed xenograft tumor growth[23–26]. In this study, we aimed to investigate the effect of ATRA on the expression of angiogenic factor receptors and tumor cell survival using EC1 cell line. We found that ATRA inhibited cell viability via cell apoptotic pathway in a RARα-dependent manner. Importantly, ATRA-reduced tumor growth was correlated with the low protein levels of angiogenic factor receptor Ang-1, Ang-2 and Tie2 in xenograft tumors of EC1 cells. Our new findings add more evidence that ATRA exerts the anti-tumorigenic effect in esophageal cancer (**Fig 6**).

## Supporting information

S1 Fig ATRA treatment shows a time-dependent and dose-dependent inhibition on cell viability. EC1 cells were cultured in RPMI-1640 supplemented with 10% FBS and seeded in 96 well plates. (A) After 0 hours, 24 hours and 48 hours, the number of live cells was counted in ATRA and fluorouracil treatment groups. (B) The proportion of dead cells were calculated and presented as Percent of death cells/total number of cells. (C) After 24 hours, ATRA and fluorouracil show additive effect on cell proliferation. (D) After 24 hours, ATRA caused apoptosis of cells. Densitometry analysis of protein levels of Cl-PARP. (E) The effect of ATRA and fluorouracil on the expression of Cl-Caspase3. (F) Densitometry analysis of protein levels of Cl-caspase3. *p<0.05; **p<0.01; *** p<0.001. Student t-test.
(XLSX)

S2 Fig ATRA treatment reduces cell migration in EC1 cells. EC1 cells were cultured in RPMI-1640 supplemented with 10% FBS and seeded in 6 well plates. Scratches on cell monolayer were made using pipette tips when cells became confluent. Cells were then treated with 3 concentrations of ATRA (0.1, 1, 10 μmol/L), fluorouracil (100 mg/L), or untreated for 24 hours. Images were chosen from 10 random fields to calculate the average distances. Data were presented as average length of cell-free void ± SD. (B) Representative pictures of wound healing assay. *p<0.05; **p<0.01; *** p<0.001. Student t-test.
(XLSX)

S3 Fig The transcript levels of Angiopioteins-Tie-2 pathway are downregulated in EC1 cells. EC1 cells were treated with ATRA at 0.1, 1, or 10 μmol/L, 100 mg/L fluorouracil, 10 μmol/L AM80, 100 mg/L fluorouracil plus 10 μmol/L AM80, or untreated for 24 hours. RNA was isolated from treated cells. Real-time RT-PCR analysis was performed to assessed the transcript levels of (A) Ang-1, Ang-2 and Tie-2. (B) Ang-1. (C) Ang-2. (D) Tie-2. (E) VEGF. (F) Flt-1. (G) KDR. *p<0.05; **p<0.01; *** p<0.001. Student t-test.
(XLSX)

S4 Fig ATRA treatment decreases the expression of Ang-1, Ang-2 and Tie-2 in EC1 cells. EC1 cells were treated with 3 concentrations of ATRA (0.1, 1, 10 μmol/L), fluorouracil (100 mg/L) for 24 hours, or untreated. (A) The protein levels of Ang-1, Ang-2 and Tie-2 were examined using western blot. Densitometry analysis of the protein levels of Ang-1, Ang-2 or Tie-2 (B); Ang-1 (C); Ang-2(D); and Tie-2 (E). β-actin was used as a loading control. *p<0.05; **p<0.01; *** p<0.001. Student t-test.
(XLSX)

S5 Fig ATRA treatment suppresses the growth of xenograft tumors of EC1 cells and improves the cachexia of mice. (A) 1x10^6^ EC1 cells were subcutaneously injected into mice at both flanks on day 0. Ten days post-cell inoculation, mice bearing xenograft tumors were randomized to five groups and treated for 10 days with placebo, fluorouracil (50 mg/kg/day), or 3 concentrations of ATRA (0.1, 1, or 10 mg/kg/day). Mice were killed on day 20. Mouse body weight was measured before and after cells implantation, also before and after treatment. (B) The cachexia was recorded in mice treated with ATRA, fluorouracil, or placebo. Cachexia was assessed by body weight loss. (C) Images of tumors isolated from mice treated with ATRA and fluorouracil. (D) Average tumor size was calculated and shown in panel C. (E) Immunohistochemical staining of CD31, Ang-1, Ang-2 and Tie-2 in subcutaneous tumors. *p<0.05; **p<0.01; *** p<0.001. Student t-test.
(XLSX)
